# Tablet-Based Sensor: A Stable and User-Friendly Tool for Point-of-Care Detection of Glucose in Urine

**DOI:** 10.3390/bios13090893

**Published:** 2023-09-19

**Authors:** Hasti Hajimiri, Seyed Hamid Safiabadi Tali, Muna Al-Kassawneh, Zubi Sadiq, Sana Jahanshahi-Anbuhi

**Affiliations:** Department of Chemical and Materials Engineering, Gina Cody School of Engineering, Concordia University, Montreal, QC H3G 2W1, Canada; hasti.hajimiri@gmail.com (H.H.); s_safiab@live.concordia.ca (S.H.S.T.); muna.kassawneh@gmail.com (M.A.-K.); zubi.sadiq@mail.concordia.ca (Z.S.)

**Keywords:** enzyme stability, tablet, biosensor, encapsulation, glucose, urine

## Abstract

The colorimetric detection of glucose in urine through enzymatic reactions offers a low-cost and non-invasive method to aid in diabetes management. Nonetheless, the vulnerability of enzymes to environmental conditions, particularly elevated temperatures, and their activity loss pose significant challenges for transportation and storage. In this work, we developed a stable and portable tablet sensor as a user-friendly platform for glucose monitoring. This innovative device encapsulates glucose oxidase and horseradish peroxidase enzymes with dextran, transforming them into solid tablets and ensuring enhanced stability and practicality. The enzymatic tablet-based sensor detected glucose in urine samples within 5 min, using 3,3′,5,5′-tetramethylbenzidine (TMB) as the indicator. The tablet sensor exhibited responsive performance within the clinically relevant range of 0–6 mM glucose, with a limit of detection of 0.013 mM. Furthermore, the tablets detected glucose in spiked real human urine samples, without pre-processing, with high precision. Additionally, with regard to thermal stability, the enzyme tablets better maintained their activity at an elevated temperature as high as 60 °C compared to the solution-phase enzymes, demonstrating the enhanced stability of the enzymes under harsh conditions. The availability of these stable and portable tablet sensors will greatly ease the transportation and application of glucose sensors, enhancing the accessibility of glucose monitoring, particularly in resource-limited settings.

## 1. Introduction

Diabetes is a chronic condition that affects millions of people worldwide. According to the Atlas of the International Diabetes Federation [1], there were approximately 537 million adults diagnosed with diabetes in 2022 worldwide, while the World Health Organization (WHO) [2] has estimated that in low- and middle-income countries, one in two adults has undiagnosed diabetes. While there are various types of diabetes, in all instances there is an abnormal accumulation of glucose (C₆H₁₂O₆) in the bloodstream [3]. If left untreated, diabetes can lead to a variety of dangerous problems such as an increased risk of cardiovascular diseases [4], heart strokes [5], kidney failures [6], blindness [7], and amputations [8]. Therefore, addressing diabetes in the early stages of its development greatly lowers the risks involved while decreasing the future healthcare costs associated with the required treatment. Consequently, there is increasing demand for non-invasive, user-friendly, and cost-effective glucose monitoring methods to assist in the early detection of this disease, which in turn would provide a chance to mitigate the adverse effects of diabetes [9].

One viable solution to deal with this demand is the use of point-of-care (POC) analytical devices [10]. Recent advances in POC testing have led to innovative solutions for sensitive analyte detection. For instance, Gao et al. [11] introduced a user-friendly POC tool for the on-site analysis of DNA adenine methyltransferase activity. Their approach employs DNA tetrahedra-based hydrogel to capture glucose-producing enzymes, streamlining target recognition and signal transduction on paper. This integration of DNA hydrogel, enzyme-encapsulated substrates, and a commercial glucose test strip exemplifies the versatility of POC devices. Another recent study by Rauf et al. [12] presented a nanostructured gold-modified Laser-Scribed Graphene electrode system offering a two-fold sensitivity boost over conventional electrodes, making it ideal for POC applications. Their work also yielded a highly sensitive aptasensor for Her-2 biomarker detection, highlighting the potential for rapid and precise POC diagnostics, contributing to the field’s evolution.

POC devices are compact, portable, and simple to operate, making them suitable for practical deployment in hospitals, clinics, and in-home use for consistent monitoring of glucose to regulate its level in blood. Currently, the most common POC method for glucose detection is enzymatic detection through electrochemical test strips utilized to measure glucose levels in blood [13]. Nonetheless, taking blood from patients is an invasive process, which presents a significant obstacle to frequent glucose monitoring [14]. For instance, this is a widely acknowledged difficulty in managing childhood-onset type 1 diabetes, where children are required to keep a check on their blood sugar levels every time they consume food [15]. In contrast, detecting glucose in urine is a non-invasive approach that can be a suitable alternative or supportive method to estimate glucose levels for diabetes management [16]. Various techniques using different POC platforms have been reported for glucose detection, including colorimetric, electrochemical, and fluorescent methods on paper-based, thread-based, and other types of microfluidic devices [17,18,19,20,21,22,23,24]. Still, these platforms are prone to limitations such as poor mixing of reagents and samples, non-uniform color formation, decreased precision, and the need for external equipment and cumbersome immobilization steps for the reagents.

To tackle the challenges encountered with other platforms, one possible approach involves adopting enzymatic detection in bulk solutions. Utilizing enzymes offers high sensitivity and specificity for the assays [25], while the solution phase allows for proper mixing and homogeneity and, in turn, better repeatability and accuracy. However, solution-based enzymatic detection still suffers from drawbacks such as the enzymes being denatured at higher temperatures and under altered pH conditions as well as the necessity for specific temperature requirements during transportation that need to be addressed [26]. In this regard, a tablet-based detection platform introduced in 2014 is a viable method that combines the advantages of the solution-based detection method and the long-term stability of solid-phase platforms [27]. In this technique, the reagents are encapsulated in polysaccharides such as pullulan and dextran to form solid tablets. The encapsulation of the bioreagents in polysaccharides enhances the stability of enzymes in different external conditions, while the solid tablets act as a portable easy-to-use detection platform that can be inserted into the sample medium and readily dissolve to initiate the assay procedure. This approach has been successfully applied to a variety of assays, including the detection of phosphate in water [28] using AuNPs and nanozyme sensors for glucose detection in saliva with pullulan-stabilized AuNP tablets [29]. Among the various materials that have been explored for enzyme encapsulation, dextran has emerged as a promising candidate. This is primarily due to its remarkable biocompatibility, biodegradability, and low toxicity [30]. Dextran is a water-soluble polysaccharide composed of glucose molecules linked by α-1,6 and α-1,3 glycosidic bonds and has been widely used in biomedical applications, such as drug delivery and tissue engineering [31].

In this work, for the first time, we developed an enzyme tablet bioassay for the non-invasive point-of-care detection of glucose in urine. Our method involved encapsulating pre-measured quantities of both glucose oxidase (GOx) and horseradish peroxidase (HRP) in dextran to produce all-in-one solid enzyme tablets. The tablets were then characterized using Fourier Transform Infrared Spectroscopy (FTIR) and Atomic Force Spectroscopy (AFM). A colorimetric assay based on enzyme tablets was then carried out using 3,3′,5,5′-tetramethylbenzidine (TMB) as the chromogenic agent, and the analytical performance of the assay was assessed in complex matrices of artificial urine and real human urine. Lastly, the stability of the enzyme tablets was studied for storage at room temperature and in accelerated stress-testing conditions at elevated temperatures.

## 2. Materials and Methods

### 2.1. Chemicals and Materials

Horseradish peroxidase (HRP, Cat. No. P8250), glucose oxidase from *Aspergillus niger* (GOx, Cat. No. G7141), glucose (C_6_H_12_O_6_, Cat. No. G8270), 3,3′,5,5′-tetramethylbenzidine (TMB, Cat. No. 860336), sodium phosphate dibasic (Na_2_HPO_4_, Cat. No. S9763), sodium phosphate monobasic monohydrate (NaH_2_-PO_4_⋅H_2_O, Cat. No. S9638), citric acid (C_6_H_8_O_7_, Cat. No. 251275), sodium hydroxide (NaOH, Cat. No. S5881), dextran (average Mw. ~100 kDa), fructose (Cat. No. PHR1002), maltose (Cat. No. PHR1497), trehalose (Cat. No. PHR1344), and HPLC water (Cat. No. 270733) were acquired from Sigma-Aldrich, Oakville, ON, Canada. Dimethyl sulfoxide (DMSO) (Cat. No. D128–1) was obtained from Fisher Scientific, Toronto, ON, Canada. To simulate realistic conditions, stabilized artificial urine (Cat. No. BZ104) was carefully sourced from Biochemazone, Leduc, AB, Canada. A non-stick carbon-steel tray (Betty Crocker, Golden Valley, MN, USA) was purchased from a local store in Canada. Real urine samples were obtained from a healthy volunteer and were used without any further treatment. This study was conducted with the agreement of Concordia University’s Institutional Review Board with the approval number SC5823 and the BioPermit number B-SJA-22–01. Also, written and oral informed consent were obtained from the volunteers.

### 2.2. Characterizations

The tablets were characterized using Fourier Transform Infrared Spectroscopy (FTIR) to study the functional groups using a Nicolet™ iS20 FTIR Spectrometer (Thermo Scientific Instrument Co., Waltham, MA, USA). Atomic Force Spectroscopy (AFM) was performed to study a tablet’s surface morphology using a TOSCA 400 AFM (Anton Paar, Graz, Austria).

### 2.3. Preparation of Enzyme Solution and Fabrication of Enzyme-Based Tablets

**Figure 1:**
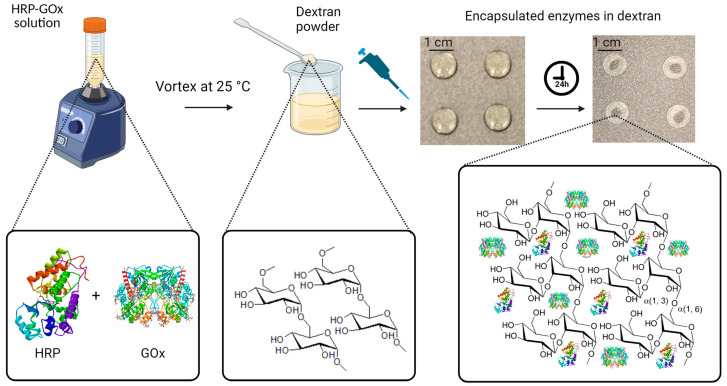
Fabrication of enzyme-based tablet sensor using dextran powder as the polysaccharide encapsulating agent. Initially, HRP and GOx were dissolved in phosphate buffer at a pH of 7.4. Then, 0.3 mL of this enzyme solution was mixed with 1.5 mL of the citrate–phosphate buffer with a pH of 5. To enable tablet formation, 6% *w*/*v* dextran was added to the mixture. In the final step, 60 μL aliquots of the resulting solution were carefully dispensed onto a non-stick tray, which was left to air dry at room temperature for 24 h, resulting in solid tablets.

First, the enzyme solution was prepared by dissolving HRP (0.15 mg mL^−1^) and GOx (180 U mL^−1^) in 20 mL of phosphate buffer (0.01 M) with a pH of 7.4. The buffer was prepared by dissolving 0.606 g of sodium phosphate dibasic and 0.101 g of sodium phosphate monobasic monohydrate in 24 mL of deionized water, followed by pH adjustment by the addition of sodium hydroxide and diluting to a final volume of 30 mL with deionized water. The pH was monitored using a pH meter device (AB200 pH/mV/conductivity, Fisher Scientific Accumet, Singapore, Singapore). For the next step, we prepared a citrate–phosphate buffer solution (0.05 M) with a pH of 5. For this purpose, 0.5445 g of sodium phosphate dibasic (0.2 M) and 0.2882 g of citric acid (0.1 M) were added to 24 mL of deionized water, followed by the addition of sodium hydroxide for pH adjustment and dilution to 30 mL by adding deionized water. Then, a mixture solution containing 0.3 mL of the enzyme solution and 1.5 mL of the citrate–phosphate buffer solution was prepared. Next, 6% *w/v* dextran was added to the mixture solution, and it was thoroughly mixed using a vortex mixer (Model# 945FIA-LUS, 50/60 Hz, Fisherbrand, Waltham, MA USA) to ensure the complete dissolution of the chemical compounds. To prepare the solid polysaccharide-encapsulated enzyme tablets, the resulting solution was pipetted onto a carbon-steel tray in 60 µL aliquots for each tablet. The aliquots were then allowed to dry on the tray for 24 h at room temperature, resulting in solid tablets. The drying process was evaluated by monitoring the weight of the aliquots, which was measured shortly after pipetting and over the 24 h of air drying. The initial weights of the aliquots were determined to be 62.26 ± 0.15 mg, which reached an average weight of 5.8 ± 0.21 mg for the formed solid tablets after fully drying. The differences in weight between the tablets before and after drying corresponded to the amount of water that evaporated during the drying process. This measurement allowed us to keep track of the water evaporation extents in order to ensure the consistency of tablet composition and properties. Figure 1 represents the fabrication procedure using dextran powder as the polysaccharide encapsulating agent and demonstrates the function of dextran long-chain polymers extending over the enzymes for better preservation and stability.

### 2.4. Detection of Glucose in Artificial Urine Using Dextran-Based Tablets

**Figure 2:**
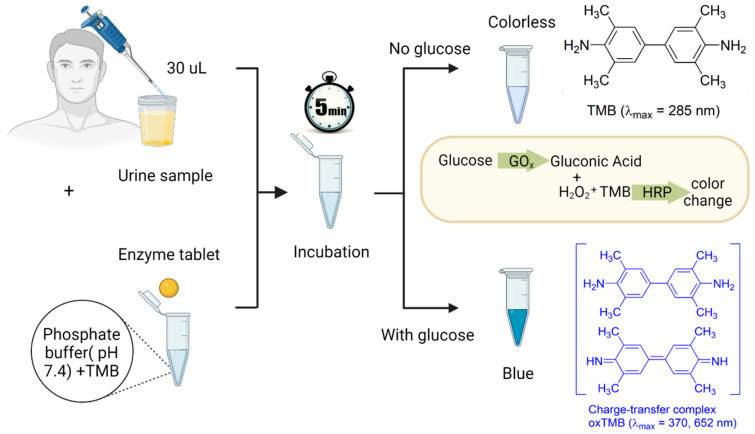
The detection procedure of glucose in urine samples. First, 30 µL of a urine sample and a single enzyme tablet are added to a test tube containing 240 µL of a phosphate buffer with a pH of 7.4 and 5 µL of the TMB solution. After 5 min of incubation, if the urine sample contains glucose, the GOx catalyzes glucose to gluconic acid and H_2_O_2_. Then, the HRP enzyme available in the tablet acts as a peroxidase enzyme and transfers electrons to the TMB substrate, which causes the color of the solution to turn blue due to the oxidation of the chromogenic TMB.

To prepare the detection system, 8.4 mg of TMB was dissolved in 1 mL of DMSO, resulting in a TMB concentration of 35 mM in DMSO. The obtained solution was kept in a fridge at 4 °C for further use. Artificial urine samples were then spiked with glucose at varying concentrations ranging from 0.1 to 5 mM. To run a detection test, 240 µL of a phosphate buffer with a pH of 7.4 was added to a 2 mL test tube pre-loaded with 5 µL of the TMB solution. This buffer served two purposes: it diluted the urine sample to ensure a suitable sensor response within the desired biologically relevant concentration range of glucose (0–6 mM) and adjusted the pH of the overall system for proper enzyme and TMB functioning. To initiate the colorimetric assay, a single tablet and 30 µL of the spiked artificial urine sample were added to the tube, as shown in Figure 2. Proper and consistent mixing can be achieved by using a vortex mixer, allowing for the uniform distribution of the components. Alternatively, the mixing process can be performed using gentle hand shaking. The final step was to allow the mixture to sit undisturbed for 5 min. During this time, the enzymatic reaction took place, leading to the development of a blueish color spectrum, corresponding to different concentrations of glucose. To develop a calibration curve and quantify the observations, the resulting color was analyzed using a UV-vis spectrometer (BioTek, Santa Clara, CA, USA, Cytation 5, imaging reader).

### 2.5. Glucose Detection in Real Human Urine

To demonstrate the practical application of our tablet sensor in real-world conditions, the detection of glucose was also conducted in real human urine samples (pH 6.5–7.0). The spiking test was performed using glucose concentrations of 1, 2, and 3 mM, and the method was validated by calculating the percent recovery (%R) and relative standard deviation (%RSD) of the results.

### 2.6. Interference Study for the Colorimetric Detection of Glucose

Potential interferants such as maltose, fructose, and trehalose were studied for our proposed glucose assay following the procedure mentioned in Section 2.4. All the interferants were used at a 10 mM concentration in artificial urine samples. A UV-vis spectrometer was used to measure the absorbance intensity of the formed blue color by obtaining the absorbance at 652 nm on a 96-well plate reader (Ultident Scientific, Saint-Laurent, QC, Canada).

### 2.7. Stability Tests

To assess the stability of the tablet sensors compared to the solution as well as their resistance to heat, both solid and liquid assays were subjected to accelerated stress-testing conditions at 60 °C for 24 h in an oven without forced-air convection (Thermo Scientific, Model# PR30525G, Waltham, MA, USA). Following this thermal exposure, colorimetric detection tests were performed using spiked artificial urine, as explained in Section 2.4. UV-vis spectroscopy was used to measure the absorbance at 652 nm over 2, 4, 6, and 24 h, and the results were compared to those of the freshly prepared enzyme tablet and solution assays.

## 3. Results and Discussion

### 3.1. Characterization of Tablets

The tablet-based sensors were developed to detect glucose in urine samples through the enzymatic reaction of the tablet components with glucose and the chromogenic substrate TMB. First, the enzyme solution was prepared by mixing dextran powder in a buffer containing GOx and HRP enzymes. Next, solid tablets were created by pipetting the dextran–enzyme solution in aliquots of 60 µL and air drying at room temperature for 24 h. The tablets were characterized using FTIR and AFM analyses to study and identify the particular functional groups of the tablets as well as to examine the physical properties including morphology, surface texture, and roughness, which have great impacts on tablet durability.

**Figure 3:**
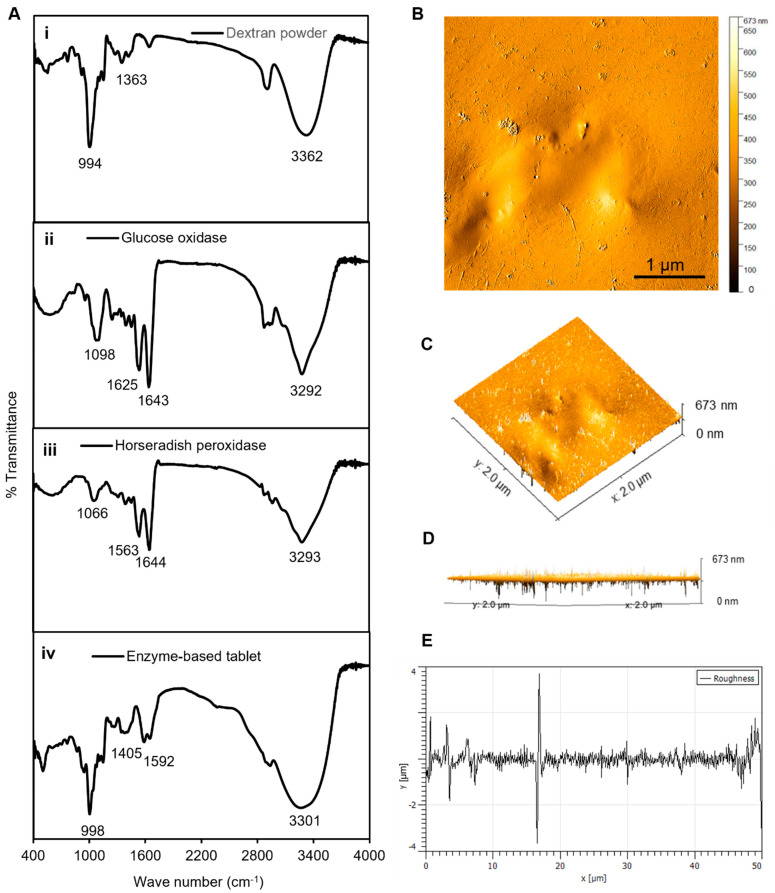
Characterization of tablets. (**A**) The FTIR analyses of (**i**) dextran powder, (**ii**) GOx, (**iii**) HRP, and (**iv**) solid tablets. (**B**) The AFM analyses of tablets, demonstrating the 2D image of the height profile in an amplitude trace; (**C**,**D**) 3D image of the height profile of (**B**); and (**E**) height distribution as surface roughness and texture description of image (**B**). The average roughness was ~1.1 μm, the average maximum height of the roughness was ~8 μm, the average maximum roughness valley depth was ~4.2 μm, the maximum peak-to-valley roughness was ~4 μm, and the waviness average was ~0.15 μm. The FTIR and AFM results confirmed a strong binding connection between the dextran polymer and the enzyme mixture in the tablet platform.

In general, enzymes contain specific functional groups that appear in the form of weak peaks or disappear in the case of losing efficiency [32,33]. Therefore, the FTIR analyses served as an appropriate method to confirm the presence of the required functional group responsible for the activity of the enzymes in our established enzyme-based tablet sensor. The FTIR analysis of pristine dextran powder indicated a peak at 3362 cm^−1^ due to the −OH group stretching vibrations, while it was shifted to 3301 cm^−1^ in the tablet. The bands at 1163, 1183, and 994 cm^−1^ corresponded to the stretching vibrations of C−O bonds, the alcoholic hydroxyl (C−OH), and α-glycosidic bonds (C−O−C) in dextran [34], respectively, as shown in Figure 3(Ai). The spectrum of GOx showed bands at 1643 and 1625 cm^−1^ (Figure 3(Aii)), which were attributed to amide I and amide II [35]. However, the bands were slightly shifted with less density in tablets to 1405 and 1592 cm^−1^. Furthermore, similar bands were observed in the spectrum of HRP, which indicated peaks between 1563 and 1644 cm^−1^ assigned to amide I (α-helix structure) [36]. Another observed spectral region of HRP was at 3293 cm^−1^, which was attributed to O–H vibration modes, as presented in Figure 3C. The presence of all these bands in the tablet indicated the successful encapsulation of both enzymes into the dextran matrix, as shown in Figure 3D.

Next, AFM analyses were performed to analyze the surface morphology of the enzymes and their dextran surroundings in a solid-state format. The results presented in Figure 3B–D show that the dextran-matrix-containing enzymes had smooth surfaces, as indicated by the phase-trace analyses. The phase-trace image in the figure revealed that the mean elevation of the tablet was between 2 and 8 nm, indicating that enzymes were well preserved throughout the exopolysaccharide substance, which resulted in a very smooth surface. Furthermore, the surface roughness can be used to determine how a solid material interacts with the surrounding environment [37]. In the case of our tablet sensor, the average roughness was ~1.1 μm, the average maximum height of the roughness was ~8 μm, the average maximum roughness valley depth was ~4.2 μm, the maximum peak-to-valley roughness was ~4 μm, and the waviness average was ~0.15 μm, as presented in Figure 3. These results of the FTIR and AFM analyses confirmed a strong binding connection between the dextran polymer and the enzyme mixture in the tablet platform, which confirmed that the enzymes were well preserved in the dextran structure, prolonging the shelf life, stability, effectivity, and performance of these tablet-based sensors.

### 3.2. Optimization of the Experimental Conditions

Before proceeding with the colorimetric detection assay, it was crucial to optimize various experimental conditions to ensure the most accurate and sensitive results. In this section, we describe the optimization of five key parameters: the TMB concentration, sample volume, enzyme concentration in tablets, pH of the system, and dextran concentration. Each parameter played a critical role in the assay’s performance, and deviations from the optimal values led to compromised accuracy or reduced sensitivity.

**Figure 4:**
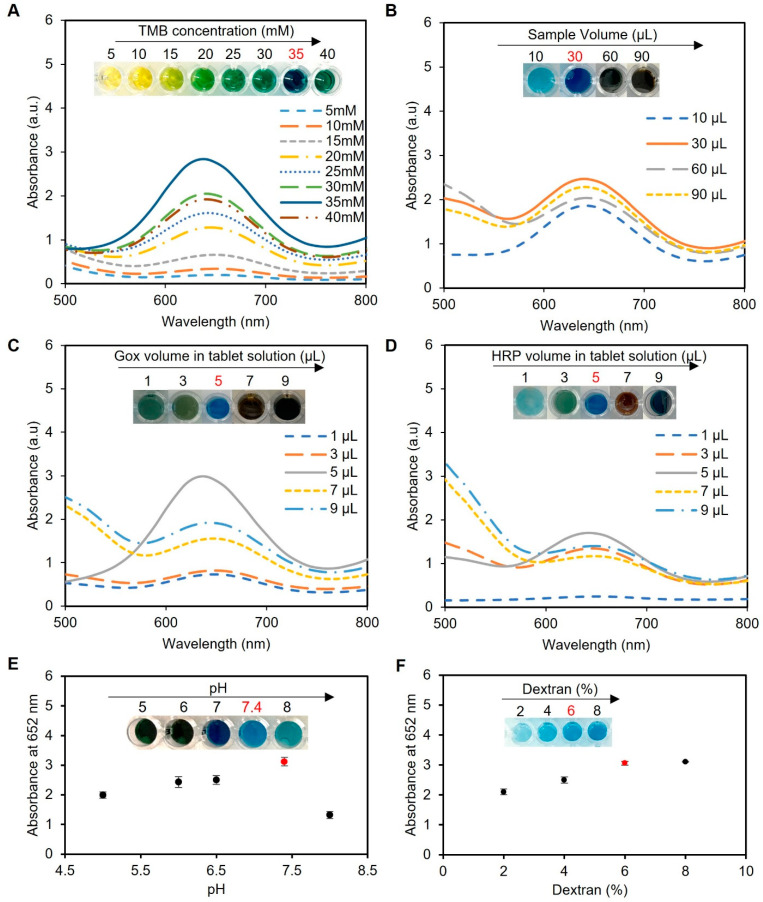
Optimization of the experimental conditions for glucose detection using enzyme-based tablets. (**A**) Optimization of the TMB concentration using various concentrations of TMB (5–40 mM). The results indicated that 35 mM TMB was the best to give the desired blue color, while concentrations from 5 to 15 mM gave a yellow color, a greenish color was obtained in the concentrations between 20 and 30 mM, and a very dark blue was observed using 40 mM TMB. (**B**) The effect of the sample volume on the blue color development. A sample volume of 30 µL produced the most intense blue color. (**C**,**D**) Optimization of enzyme concentration in tablets. The enzyme ratio was carefully optimized using 5 µL of HRP and 5 µL of GOx in 50 µL of citrate–phosphate buffer (pH 5) to prepare the solution of one tablet. This ratio achieved the desired blue color without excess accumulation or incomplete oxidation of TMB. (**E**) pH optimization of the system. The pH of the reaction mixture was investigated, and a pH of 7.4 was identified as the optimal condition for the system. (**F**) Optimization of dextran concentration for better enzyme encapsulation. The results indicated that a 6% *w/v* concentration effectively preserved enzymatic activity and led to the desired blue color development. Lower concentrations (2% and 4% *w*/*v*) compromised enzymatic activity, while an 8% *w/v* concentration increased viscosity, making the solution challenging to handle during the pipetting and drying processes. Therefore, a 6% dextran *w/v* solution was used to create the tablet sensor used throughout the study.

The TMB concentration played a crucial role in the colorimetric detection assay, as it directly influenced the formation of the blue-colored product through oxidation by HRP in the presence of H_2_O_2_. Excessive TMB concentrations led to over-oxidation, resulting in a black-blue color [38] and causing the signal to saturate and give false readings, while a low TMB concentration resulted in a weak signal and decreased assay sensitivity. To determine the ideal TMB concentration, we conducted experiments using a range of TMB concentrations from 5 to 40 mM dissolved in DMSO. The sample utilized in these experiments consisted of artificial urine spiked with 2 mM glucose, with a total working solution containing 60 µL of the sample, 200 µL of phosphate buffer (pH 7.4), and one tablet sensor. Our investigations revealed that a TMB concentration of 35 mM provided the best blue color, as presented in Figure 4A, indicating its suitability for use in the assay.

Next, the sample volume of the assay protocol was optimized, as presented in Figure 4B. We observed that a low sample volume (10 µL) resulted in low color intensity and thus less sensitivity, while high sample volumes (60 and 90 µL) resulted in color saturation and compromised the working range of the assay. We found that a sample volume of 30 µL produced the most intense blue color when using one tablet sensor and adding 5 mM glucose to 200 µL of phosphate buffer (pH 7.4) and 5 µL of TMB at a concentration of 35 mM, indicating its suitability for use in the assay.

Optimization of the enzyme ratios was also performed using a sample of water spiked with glucose (5 mM), totaling 60 µL, along with 200 µL of phosphate buffer (pH 7.4), 5 µL of TMB at a concentration of 35 mM, and one tablet. Our investigation revealed that a ratio of 5 µL of 0.15 mg mL^−1^ HRP to 5 µL of 180 U mL^−1^ GOx in 50 µL of 0.05 M citrate–phosphate buffer (pH 5) produced the desired blue color, as shown in Figure 4C,D, without excess TMB accumulation (black-blue color) or incomplete oxidation (faint blue color).

Next, we optimized the pH of the reaction mixture since it showed a significant impact on the rate of TMB oxidation by HRP and, in turn, the assay results. In our experiments, we utilized a sample consisting of water spiked with 5 mM glucose, with a total sample volume of 60 µL; 200 µL of phosphate buffer; 5 µL of TMB at a concentration of 35 mM; and one tablet sensor. Our findings indicated that the optimal pH for the HRP/TMB system was 7.4, as pH values outside of this range resulted in weaker signals or undesirable color changes in the final product (Figure 4E). Therefore, a significant decline in the effectiveness of producing blue-colored products occurred when the pH of the samples was below or exceeded 7.4 due to the comparatively elevated redox potential of the substrate system, which led to a decreased vulnerability to oxidation [39].

The concentration of dextran, utilized as an encapsulating agent in the fabrication of tablets, is a crucial factor that influences the preservation and performance of the enzymes. In this optimization study, different concentrations of dextran (2, 4, 6, and 8% *w*/*v*) were tested in solution and tablet formats. Our findings revealed that a dextran concentration of 6% *w/v* provided the optimal conditions for preserving the enzymatic activity, resulting in the desired blue color development, as depicted in Figure 4F. However, lower concentrations of dextran (2% and 4% *w*/*v*) failed to adequately preserve the enzymes, leading to reduced enzymatic activity and compromised colorimetric detection performance. On the other hand, while a dextran concentration of 8% *w/v* successfully preserved the enzymatic activity, it caused the solution to become excessively thick and viscous. This increased viscosity made it challenging to handle the solution during the pipetting and drying process, affecting the uniform distribution of the solution and impeding the subsequent drying process. Similar results were observed when using the solution format without drying to create tablets when the same concentrations of dextran were used.

**Figure 5:**
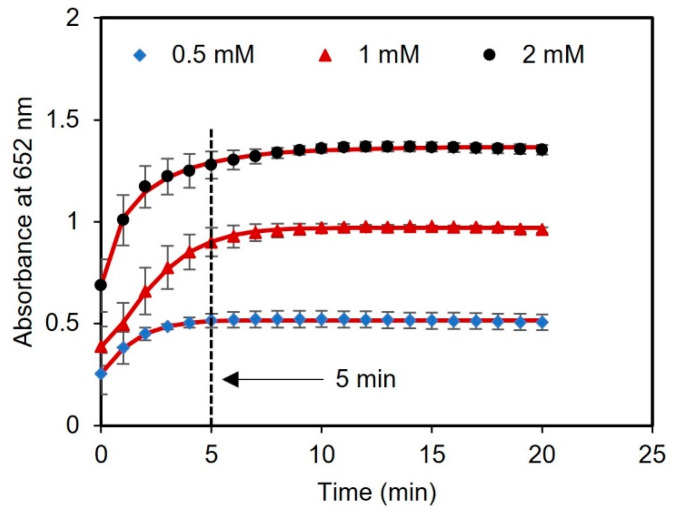
Kinetic analysis using tablets. The time duration for glucose detection using concentrations of 0.5, 1, and 2 mM was found to be 5 min.

Next, we performed a kinetic study with different concentrations of glucose (0.5, 1, and 2 mM) to evaluate the optimal time for reading the results. The time course of light absorbance was measured for three samples spiked with glucose using a UV-vis spectrometer. The absorbance at 652 nm was measured at 1 min intervals over a total time of 20 min. After 5 min of reagent incubation, there were no significant changes in the absorbance of the samples, indicating that the sensor response had stabilized and reached saturation, as presented in Figure 5. Therefore, 5 min was chosen as the optimal time to measure the readings of the assay after adding the tablet sensor to the samples.

Overall, the best experimental conditions to show superlative tablet sensor performance with higher blue color formation were at a pH of 7.4 using 35 mM TMB, a 30 µL sample volume, 5 µL of 180 U mL^−1^ GOx, 5 µL of 0.15 mg mL^−1^ HRP, and 6% dextran (*w*/*v*) with result reading after 5 min. Therefore, these conditions were used in the following tests to detect glucose in artificial and human urine samples.

### 3.3. Analytical Performance

**Figure 6:**
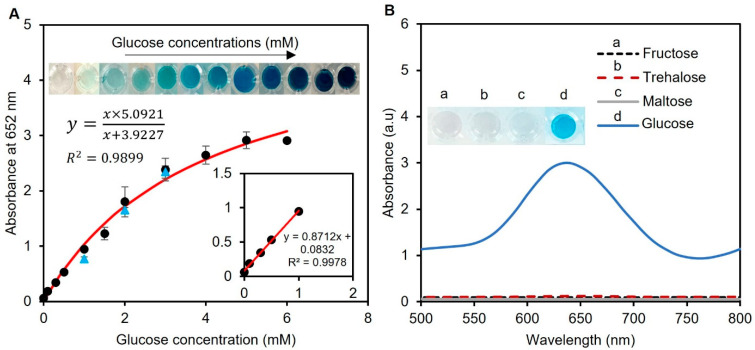
Analytical performance of the assay and interference study. (**A**) Glucose concentrations between 0.1 and 6 mM were used to create the calibration curve. The curve was found to follow the Michaelis–Menten equation with an R^2^ > 0.98, showing the compatibility of the data with a standard saturation model. The inset shows the linear range of the detection system between 0 and 1 mM. Real human urine samples were also tested with spiked glucose concentrations of 1, 2, and 3 mM (blue triangle points). Each data point is the mean ± the standard deviation of three replications. (**B**) The potential interferants of glucose were tested using 10 mM fructose, trehalose, and maltose. While the working solution remained colorless for all three sugars, a glucose sample of 0–6 mM was able to turn the solution blue, indicating the sensor’s specificity towards glucose detection.

The analytical performance of the enzyme-tablet colorimetric detection system was evaluated by generating a calibration curve by adding tablets to samples of known concentrations of spiked artificial urine containing glucose. This approach allowed us to measure the correlation between the glucose concentration and absorbance at 652 nm, which was proportional to the amount of glucose present in the sample. The Michaelis–Menten equation was used to plot the relationship between the glucose concentration in the samples and absorbance, which resulted in a calibration curve with an R^2^ value of 0.9899, as presented in Figure 6A. This value indicates a strong correlation between the glucose concentration and absorbance and suggests that our system’s results fit well with a typical saturation model. Also, the working range of our detection system was found to be 0–6 mM in artificial human urine samples, which is the clinically relevant range of glucose concentration found in human urine [40]. Furthermore, we determined the limit of detection for our system using the formula 3σ/μ, where σ and μ represent the relative standard deviation and slope of the linear calibration plot, respectively. Our system was found to have a limit of detection of 0.013 mM, which is a highly sensitive detection threshold compared to other reported results in the literature [41,42,43]. Thus, our system can detect glucose at very low concentrations, which expands the sensor’s applications in clinical settings.

To evaluate the practical suitability of our suggested tablet sensor, glucose detection was also accomplished in human urine samples. Three human samples were collected and used without any previous treatments. The samples were spiked with 1, 2, and 3 mM glucose, and the tests were measured at 652 nm using UV-vis spectroscopy with three replicas, as presented in Figure 6A with blue triangles. The validity of our tablet sensor was confirmed by the percent recovery (%R) and percent relative standard deviation (%RSD) values of these samples. The %R provides an estimation of the proportion of the original substance that is retrieved in a solution, while the %RSD helps to analyze the precision of a method by calculating the dispersion of values around a mean. The %R values were found to be 81 ± 2.6, 91 ± 1.5, and 98 ± 2.2%, while the %RSD was found to be less than 3% for all replicas. These calculated %R and %RSD values of the spiked samples showed the applicability of our sensor for glucose detection in human urine in real-world applications.

### 3.4. Interference Study Tests

After assessing the analytical performance of our detection system, to understand whether other sugar compounds interfere with our glucose detection system, we investigated the impact of common sugars, such as fructose, maltose, and trehalose, on the enzymatic assay of the spiked artificial urine samples. To assess potential interference, we mixed one tablet sensor with 20 µL of 10 mM fructose, maltose, and trehalose in 240 µL of phosphate buffer (pH 7.4). The results revealed that no color was produced for all three sugars, while the system turned blue in the presence of glucose (Figure 6B). This is an important finding, as the presence of interfering substances in biological samples can lead to inaccurate results. Therefore, the ability of the developed enzymatic assay to work with high specificity towards glucose is promising for its potential application in the detection of glucose in urine samples.

### 3.5. Stability Tests

**Figure 7:**
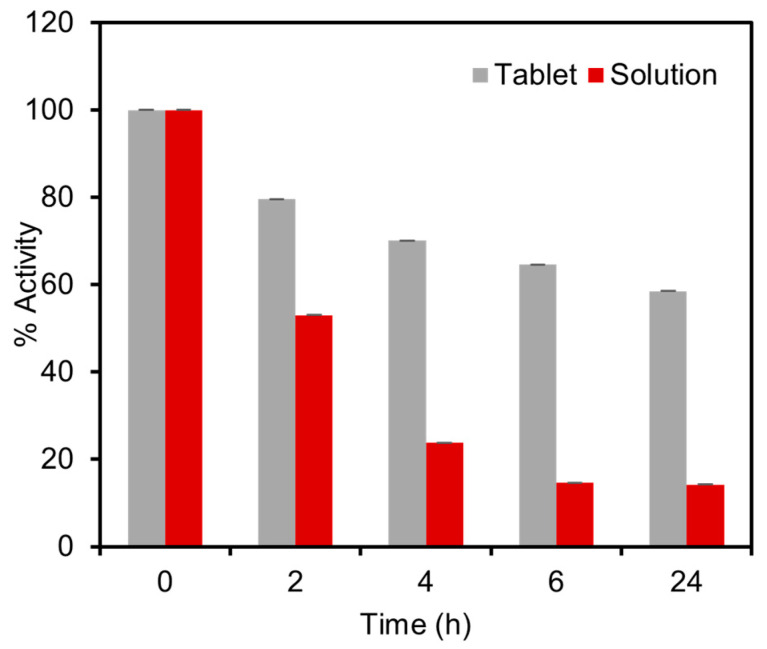
Stability tests of the enzymatic tablet-based assay under stress-testing conditions at 60 °C compared with the similar enzymatic assay stored in the solution phase. The thermal activity test was carried out by comparing the activity of tablet and solution-based sensors over a duration of 24 h. The solution exhibited a loss of 85.8% of enzymatic activity, whereas the tablets demonstrated a lower activity loss of only 41.5%.

To assess the stability of the tablets and resistance to elevated temperatures, the performance of the tablets was compared with the solution reagents under accelerated stress-testing conditions. For this experiment, both the solid tablets and the solutions were subjected to a temperature of 60 °C for a duration of 24 h, followed by colorimetric detection tests using 1.5 mM glucose in spiked artificial urine. The results revealed a significant difference in enzyme activity between the solution and the tablets after the thermal exposure procedure. The solution exhibited a considerable loss of 86% of enzymatic activity, whereas the tablets experienced a lower activity loss of only 41% in 24 h, as presented in Figure 7. To further evaluate the stability of the tablets, we conducted storage tests at room temperature. The results revealed that the tablets retained 90% and 78% of their activity after two and four weeks, respectively. These findings demonstrated that the developed tablets suitably maintain their activity for up to one month at room temperature and exhibit superior thermal stability compared to the solution-based platforms. The remarkable stability of these tablets ensures the reliability of the enzymatic assay and facilitates their storage and transportation to remote locations, even by non-experts, in a cost-effective manner.

It is also worth noting that alternative techniques such as freeze drying, which have the potential to enhance stability, would result in a powdered form. However, a powdered form can be more challenging to handle and less user-friendly compared to the solidified tablets, which provide stable reagents in pre-measured quantities.

## 4. Conclusions

This study presents a novel approach for detecting glucose in human urine samples, offering notable advantages over traditional solution-based enzymatic detection methods. The encapsulation of GOx and HRP enzymes using 6% *w*/*v* dextran was used to enhance the stability of the enzymes while providing portable solid tablets suitable for point-of-care detection systems. The detection procedure involved inserting the tablet sensor into urine samples, followed by colorimetric analysis after 5 min. The encapsulation of all-in-one pre-measured concentrations of enzymes in each tablet simplified the detection procedure and eliminated the need for measuring equipment. The developed tablet system exhibited an LoD of 0.013 mM and a working range of 0–6 mM, covering the clinically relevant range of glucose concentrations found in human urine. The assay demonstrated excellent precision, with a %RSD value of less than 3% for all samples. The recovery percentage (%R) values of 81 ± 2.6, 91 ± 1.5, and 98 ± 2.2 further validated the assay’s accuracy with real human urine samples, without the need for pre-processing steps. The interference study revealed that the developed enzymatic assay has high specificity towards glucose, showing no response for other potential interferent sugars such as maltose, trehalose, and fructose. Stability studies further confirmed that the developed enzyme-based tablet sensors have a higher stability under accelerated stress-testing conditions at 60 °C compared to solution-based enzymes. This exceptional stability facilitates storage, transportation, and practical applications, particularly outside of laboratory environments. As our research has demonstrated, optimizing the detection workflow for end users is crucial for the advancement of diagnostic technologies. To continue building upon these findings and further enhance the user experience, it is worth exploring innovative approaches. One promising avenue for future work is the potential inclusion of other key reagents such as TMB and DMSO in solid tablets. This approach could further simplify the detection process, reducing the reliance on liquid solutions and intricate handling steps. By encapsulating these reagents in solid tablets, we can potentially enhance the portability, stability, and user-friendliness of diagnostic assays. While challenges in formulation and compatibility may arise, the potential benefits in terms of accessibility and efficiency make this an avenue worthy of thorough investigation. Therefore, we encourage researchers in this field to consider this novel direction for future studies, as it holds promise for advancing diagnostic techniques and ultimately improving healthcare outcomes.

## Data Availability

The data presented in this study are available within this article.

## References

[1] International Diabetes Federation IDF Diabetes Atlas 2022 Reports. https://www.idf.org/.

[2] (2016). The mysteries of type 2 diabetes in developing countries. Bull. World Health Organ..

[3] Gilnezhad J., Firoozbakhtian A., Hosseini M., Adel S., Xu G., Ganjali M.R. (2023). An enzyme-free Ti_3_C_2_/Ni/Sm-LDH-based screen-printed-electrode for real-time sweat detection of glucose. Anal. Chim. Acta.

[4] Strain W.D., Paldanius P.M. (2018). Diabetes, cardiovascular disease and the microcirculation. Cardiovasc. Diabetol..

[5] Tun N.N., Arunagirinathan G., Munshi S.K., Pappachan J.M. (2017). Diabetes mellitus and stroke: A clinical update. World J. Diabetes.

[6] Neuen B.L., Young T., Heerspink H.J.L., Neal B., Perkovic V., Billot L., Mahaffey K.W., Charytan D.M., Wheeler D.C., Arnott C. (2019). SGLT2 inhibitors for the prevention of kidney failure in patients with type 2 diabetes: A systematic review and meta-analysis. Lancet Diabetes Endocrinol..

[7] Wykoff C.C., Khurana R.N., Nguyen Q.D., Kelly S.P., Lum F., Hall R., Abbass I.M., Abolian A.M., Stoilov I., To T.M. (2021). Risk of Blindness Among Patients with Diabetes and Newly Diagnosed Diabetic Retinopathy. Diabetes Care.

[8] Sloan G., Selvarajah D., Tesfaye S. (2021). Pathogenesis, diagnosis and clinical management of diabetic sensorimotor peripheral neuropathy. Nat. Rev. Endocrinol..

[9] Wu H., Lee C.-J., Wang H., Hu Y., Young M., Han Y., Xu F.-J., Cong H., Cheng G. (2018). Highly sensitive and stable zwitterionic poly(sulfobetaine-3,4-ethylenedioxythiophene) (PSBEDOT) glucose biosensor. Chem. Sci..

[10] Qin J., Wang W., Gao L., Yao S.Q. (2022). Emerging biosensing and transducing techniques for potential applications in point-of-care diagnostics. Chem. Sci..

[11] Gao X., Li X., Sun X., Zhang J., Zhao Y., Liu X., Li F. (2020). DNA Tetrahedra-Cross-linked Hydrogel Functionalized Paper for Onsite Analysis of DNA Methyltransferase Activity Using a Personal Glucose Meter. Anal. Chem..

[12] Rauf S., Lahcen A.A., Aljedaibi A., Beduk T., Filho J.I.d.O., Salama K.N. (2021). Gold nanostructured laser-scribed graphene: A new electrochemical biosensing platform for potential point-of-care testing of disease biomarkers. Biosens. Bioelectron..

[13] Lee H., Hong Y.J., Baik S., Hyeon T., Kim D.-H. (2018). Enzyme-Based Glucose Sensor: From Invasive to Wearable Device. Adv. Healthc. Mater..

[14] Karim N., Anderson S.R., Singh S., Ramanathan R., Bansal V. (2018). Nanostructured silver fabric as a free-standing NanoZyme for colorimetric detection of glucose in urine. Biosens. Bioelectron..

[15] Orenius T., Psych L., Säilä H., Mikola K., Ristolainen L. (2018). Fear of Injections and Needle Phobia Among Children and Adolescents: An Overview of Psychological, Behavioral, and Contextual Factors. SAGE Open Nurs..

[16] Lee T., Kim I., Cheong D.Y., Roh S., Jung H.G., Lee S.W., Kim H.S., Yoon D.S., Hong Y., Lee G. (2021). Selective colorimetric urine glucose detection by paper sensor functionalized with polyaniline nanoparticles and cell membrane. Anal. Chim. Acta.

[17] Zhang W.-Y., Zhang H., Yang F.-Q. (2022). An Economical and Portable Paper-Based Colorimetric Sensor for the Determination of Hydrogen Peroxide-Related Biomarkers. Chemosensors.

[18] Amor-Gutiérrez O., Costa-Rama E., Fernández-Abedul M.T. (2022). Paper-Based Enzymatic Electrochemical Sensors for Glucose Determination. Sensors.

[19] Akyazi T., Basabe-Desmonts L., Benito-Lopez F. (2018). Review on microfluidic paper-based analytical devices towards commercialisation. Anal. Chim. Acta.

[20] Teymourian H., Barfidokht A., Wang J. (2020). Electrochemical glucose sensors in diabetes management: An updated review (2010–2020). Chem. Soc. Rev..

[21] Weng X., Kang Y., Guo Q., Peng B., Jiang H. (2019). Recent advances in thread-based microfluidics for diagnostic applications. Biosens. Bioelectron..

[22] Ngo Y.-L.T., Nguyen P.L., Jana J., Choi W.M., Chung J.S., Hur S.H. (2021). Simple paper-based colorimetric and fluorescent glucose sensor using N-doped carbon dots and metal oxide hybrid structures. Anal. Chim. Acta.

[23] Mathur A., Nayak H.C., Rajput S., Roy S., Nagabooshanam S., Wadhwa S., Kumar R. (2021). An Enzymatic Multiplexed Impedimetric Sensor Based on α-MnO_2_/GQD Nano-Composite for the Detection of Diabetes and Diabetic Foot Ulcer Using Micro-Fluidic Platform. Chemosensors.

[24] Tali S.H.S., Hajimiri H., Sadiq Z., Jahanshahi-Anbuhi S. (2023). Engineered detection zone to enhance color uniformity on paper microfluidics fabricated via Parafilm®-heating-laser-cutting. Sens. Actuators B Chem..

[25] Feng Y., Xu Y., Liu S., Wu D., Su Z., Chen G., Liu J., Li G. (2022). Recent advances in enzyme immobilization based on novel porous framework materials and its applications in biosensing. Coord. Chem. Rev..

[26] Sellami K., Couvert A., Nasrallah N., Maachi R., Abouseoud M., Amrane A. (2022). Peroxidase enzymes as green catalysts for bioremediation and biotechnological applications: A review. Sci. Total. Environ..

[27] Jahanshahi-Anbuhi S., Pennings K., Leung V., Liu M., Carrasquilla C., Kannan B., Li Y., Pelton R., Brennan J.D., Filipe C.D.M. (2014). Pullulan Encapsulation of Labile Biomolecules to Give Stable Bioassay Tablets. Angew. Chem. Int. Ed..

[28] Esfahani A.R., Sadiq Z., Oyewunmi O.D., Tali S.H.S., Usen N., Boffito D.C., Jahanshahi-Anbuhi S. (2021). Portable, stable, and sensitive assay to detect phosphate in water with gold nanoparticles (AuNPs) and dextran tablet. Analyst.

[29] Al-Kassawneh M., Sadiq Z., Jahanshahi-Anbuhi S. (2022). Pullulan-stabilized gold nanoparticles tablet as a nanozyme sensor for point-of-care applications. Sens. Bio-Sens. Res..

[30] Lakshmi Bhavani A., Nisha J. (2010). Dextran-The Polysaccharide with Versatile Uses. Int. J. Pharma. Bio. Sci..

[31] Hu Q., Lu Y., Luo Y. (2021). Recent advances in dextran-based drug delivery systems: From fabrication strategies to applications. Carbohydr. Polym..

[32] Errico S., Moggio M., Diano N., Portaccio M., Lepore M. (2023). Different experimental approaches for Fourier-transform infrared spectroscopy applications in biology and biotechnology: A selected choice of representative results. Biotechnol. Appl. Biochem..

[33] Kumar S., Barth A. (2010). Following Enzyme Activity with Infrared Spectroscopy. Sensors.

[34] Sadiq Z., Tali S.H.S., Jahanshahi-Anbuhi S. (2022). Gold Tablets: Gold Nanoparticles Encapsulated into Dextran Tablets and Their pH-Responsive Behavior as an Easy-to-Use Platform for Multipurpose Applications. ACS Omega.

[35] Sharma B., Mandani S., Sarma T.K. (2014). Enzymes as bionanoreactors: Glucose oxidase for the synthesis of catalytic Au nanoparticles and Au nanoparticle–polyaniline nanocomposites. J. Mater. Chem. B.

[36] Tavares T.S., da Rocha E.P., Nogueira F.G.E., Torres J.A., Silva M.C., Kuca K., Ramalho T.C. (2020). Δ-FeOOH as Support for Immobilization Peroxidase: Optimization via a Chemometric Approach. Molecules.

[37] Mei S., Yang L., Pan Y., Wang D., Wang X., Tang T., Wei J. (2018). Influences of tantalum pentoxide and surface coarsening on surface roughness, hydrophilicity, surface energy, protein adsorption and cell responses to PEEK based biocomposite. Colloids Surf. B Biointerfaces.

[38] Tummala S., Bandi R., Ho Y.-P. (2022). Synthesis of Cu-doped carbon dot/chitosan film composite as a catalyst for the colorimetric detection of hydrogen peroxide and glucose. Microchim. Acta.

[39] Drozd M., Pietrzak M., Parzuchowski P.G., Malinowska E. (2016). Pitfalls and capabilities of various hydrogen donors in evaluation of peroxidase-like activity of gold nanoparticles. Anal. Bioanal. Chem..

[40] Moodley N., Ngxamngxa U., Turzyniecka M.J., Pillay T.S. (2015). Historical perspectives in clinical pathology: A history of glucose measurement. J. Clin. Pathol..

[41] Huang C., Hao Z., Wang Z., Zhao X., Wang H., Li F., Liu S., Pan Y. (2022). A fully integrated graphene-polymer field-effect transistor biosensing device for on-site detection of glucose in human urine. Mater. Today Chem..

[42] Janmee N., Preechakasedkit P., Rodthongkum N., Chailapakul O., Potiyaraj P., Ruecha N. (2021). A non-enzymatic disposable electrochemical sensor based on surface-modified screen-printed electrode CuO-IL/rGO nanocomposite for a single-step determination of glucose in human urine and electrolyte drinks. Anal. Methods.

[43] Prasad S.N., Weerathunge P., Karim N., Anderson S., Hashmi S., Mariathomas P.D., Bansal V., Ramanathan R. (2021). Non-invasive detection of glucose in human urine using a color-generating copper NanoZyme. Anal. Bioanal. Chem..

